# Reverse phonation -physiologic and clinical aspects of this speech voice therapy modality

**DOI:** 10.1016/S1808-8694(15)31077-6

**Published:** 2015-10-22

**Authors:** Leila Susana Finger, Carla Aparecida Cielo

**Affiliations:** 1 M.S. in Human Communications Disorders UFSM/RS, Speech and Hearing Therapist, Capes Scholarship holder. Mailing Address: Leila Susana Finger - R. Angelo Uglione 1645/302 Centro Santa Maria RS 97010-570.; 2 PhD in Applied Linguistics - PUC-RS, Speech and Hearing Therapist. Adjunct Professor - Department of Speech and Hearing Therapy - UFSM. Federal University of Santa Maria.

**Keywords:** voice, speech, language and hearing sciences

## Summary

Reverse phonation is the voice production during inspiration, accomplished spontaneously in situations such as when a person sighs. **Aim:** to do a literature review, describing discoveries related to the use of the reverse phonation in the clinical practice, the anatomy and physiology of its production and its effects in vocal treatments; and moreover, indications and problems of the technique for speech disorders treatment and voice enhancement. **Results:** there were reports of significant changes in vocal treatment during with the use of reverse phonation: ventricular distention, ventricular folds separation, increase in the fundamental frequency, mucous wave inverse movement; and it also facilitates the dynamic study of the larynx when associated with endoscopy, making it possible to have a better definition of lesion localization in vocal folds superficial lamina propria layers. **Conclusion:** There are few studies describing larynx behavior during reverse phonation and, for this technique to be used in a more precise and objective way, more studies are necessary in order to prove its effectiveness in practical matters.

## INTRODUCTION

Scientific studies on vocal rehabilitation started on the 30's, but it was only recently that there has been an increase in research in this field; thus, creating more scientific knowledge about vocal therapeutic approaches^1^.

The vocal approach is one therapeutic tool able to change voice patterns, aiming at obtaining the best possible voice pattern for the dysphonic patient.

Phonation competence techniques try to promote a primary muscle adjustment, so as to produce a balanced voice. This phonation method encompasses a number of techniques, among which there is reverse phonation^2^,^3^.

The present investigation aims at describing the reverse phonation vocal technique by means of a critical review that is commented on the literature, approaching the anatomophysiology of its production and its effects on the vocal tract, as well as indications and contraindications of such technique in the speech therapy clinical practice.

## MATERIALS AND METHODS

We carried out a bibliographic survey, without a limiting date, using books, journals and the Internet. For search pattern, we used the following terms: reverse phonation, inspiratory phonation, inhaling phonation and inverse phonation.

### Reverse Phonation

Reverse phonation is also called inspiratory phonation, inhaling phonation and inverted phonation^2^,^4^, ^5^, ^6^. From now on, we shall use the term reverse phonation for the present investigation.

Reverse phonation, initially described by Powers, Holtz & Ogura^7^, by means of a larynx radiologic analysis, caused by the pressure generated above the glottis, through the turbulent air inflow, adducting the vocal folds. During this maneuver, there is a ventricle relaxation and a broadening of the laryngeal vestibule, as well as glottic adduction across the board^1^, ^2^, ^3^,^8^, ^9^, ^10^, ^11^, stretching the vocal folds^2^,^9^. This maneuver is atypical in the pneumophonic engram used in habitual speech, when there is a voluntary predominance of phonation muscle action and the closure of the glottis observed during regular phonation^5^,^12^.

Reverse phonation is related to voice production during air inflow to the lungs. This happens naturally in different situations, including laugh, cry and sighs^13^. Behlau & Pontes^14^ consider reverse phonation, whistling, laugh and blowing as the best maneuvers to loose the supraglottic constriction, since they require muscle movements that do not favor vestibular fold approximation.

Lehman8 reports that, during reverse phonation, the vocal folds come together and close the glottis, as it happens during expiratory phonation. During vocal fold adduction, there is an increase in supraglottic pressure, distending the ventricles.

In reverse phonation, the sound comes from the broad and synchronic vibration of the mucosa in the opposite direction, in agreement with gravity. Supraglottic structures are called into action, there is a reduction in median and antero-posterior constriction^2^,^4^,^7^,^15^.

The vocal fold contact coefficient tends to significantly reduce during reverse phonation when compared to expiratory phonation^10^,^12^,^16^, when there also is an increase in glottic gap^9^,^10^.

The findings from Orlikoff, Baken & Kraus^10^ show that a vocal fold contact coefficient reduction was more significant in men during the utterance of the /a/ vowel. In women, no significant differences were found in the vocal fold contact coefficient^12^.

During reverse phonation, there is a significant increase in disturbance and contact instability between the vocal folds^10^,^16^. This instability was significant in the findings from Kelly & Fisher^12^, since many participants performed emissions in reverse phonation, similar to the basal sound quality.

The study from Orlikoff, Baken & Kraus^10^ showed that the quadrangular membrane seemed elongated, and it is speculated that this happens because of a larynx caudal shifting. In the study by Kelly & Fisher^12^, this elongation seems to be related to the opening in the supraglottic area, which includes the epiglottis anterior movement, which pulls or elongates the quadrangular membrane.

Kelly & Fisher^12^ also mention an arytenoid compression reduction, which do not come in contact during reverse phonation. This posterior glottis opening, during reverse phonation, is different from the opening that happens during inspiration, when vocal fold adduction varies according to the inspiratory demand^9^.

The reverse phonation technique helps aphonic patients, or those that develop some atypical form of vocalization, such as ventricular phonation, to activate the true vocal folds vibration. Those individuals that present these alterations for any given time lose their capacity to start vocalization using their true vocal folds^2^.

The current literature suggests a reduction in ventricular fold action during reverse phonation^2^,^4^,^10^,^17^,^18^. These findings come from subjective observations; however, the study from Kelly and Fisher^12^ seem to prove this statement based on objective measurements.

Behlau et al.^6^ explain that in cases in which the patient resists a clean utterance from reverse phonation, by means of sustaining a prolonged vowel, regardless of vocal quality, the patient is asked to utter small sound units, as if the patient were panting, with the inspiratory “ihn” and a short expiratory vowel, preferable the “a”, which, generally, the patients reports to be more comfortable, or even “i” or “u”, because tongue elevation in these vowels helps in removing the epiglottis from the larynx lumen.

### Reverse phonation in children

Reverse phonation is found in babies, in the first minutes of life. The inspiratory cry represents an obstruction in the breathing inspiratory phase and, after this cry, the children need additional air to fill up their lungs and stabilize breathing, which explains the latency between the end of reverse phonation and the later organic breathing. This phenomenon is associated with vocal fold adduction during reverse phonation, which requires a time for them to return to their position on the expiratory phonation onset^13^.

According to Grau, Robb & Cacace^13^, both the frequency and duration of the cry in reverse phonation are fundamentally determined by the anatomy of the child's vocal tract, which is made up of cartilage and flexible ligaments. Between four and six months of age, considerable changes start to take place in the child's vocal tract - the larynx descends down the neck. Thus, around six months of age, there is a reduction in episodes of reverse phonation cries.

### Intensity and frequency

During reverse phonation, the fundamental frequency increases significantly^2^,^10^,^12^,^13^,^16^ when compared to its expiratory counterpart. In average, the reverse phonation is 74 Hz higher^16^. On the other hand, patients with spasmodic dysphonia have a reduction in fundamental frequency during reverse phonation^9^.

Reverse phonation has a variable effect when concerning the production of different vowels. The acoustic characteristics of vowels /i, u, a/ produced by women and young men during normal phonation, when compared to the same vowels produced during reverse phonation, reveals a mean fundamental frequency, significantly higher in reverse phonation^16^.

Significant resonance differences were found in the utterance of vowel /i/ between reverse phonation and normal phonation. In uttering the vowel /u/, the fundamental frequency was considerable higher during reverse phonation. However, in uttering the vowel /a/ in reverse phonation, it was significantly lower. These results show the difference in speech mechanism articulatory control during reverse phonation when compared to normal phonation^16^.

Intensity does not vary uniformly if we compare reverse phonation emissions with those in the expiratory phase^12^. The major respiratory effort and the low adduction levels during reverse phonation also result in small intensity changes, if compared. This fact may be explained by the individual strategies used to change from expiratory to reverse phonation^12^.

### Risks involved with this technique

The cost-benefit ratio of using reverse phonation in the clinical practice must be carefully analyzed. Although this technique helps in the diagnosis of mass lesions and facilitates vocal production and training, one must take into account the possible risks, such as drying of the vocal tract after long periods of reverse phonation caused by the oral inhalation of air^16^. Besides drying, its use may cause vocal fold hematoma, if they are mobile^19^.

During prolonged reverse phonation, patients usually feel that their throats are dry and they need to clear it with one or two coughs in 5 to 10 minute intervals^9^.

According to Harrison^9^, there is no inconveniency in the use of reverse phonation during feeding, since there are no episodes of food aspiration described in the literature.

### Use of the technique in therapy

The reverse phonation technique is used in vocal rehabilitation for those cases of psychogenic aphonya, puberphonias, mid-posterior triangular glottic slit, slit by vocal fold paresis or paralysis^2^,^5^,^6^,^20^,^21^, phonation with vestibular folds and aryepiglottic phonation^2^,^5^,^6^,^22^, ^23^, ^24^, ^25^, ^26^. Many authors indicate this technique in the treatment of spasmodic dysfunctions, such an alternative source of phonation, not requiring the use of physiologic phonation, in other words, the expiratory phonation^1^, ^2^, ^3^, ^4^, ^5^, ^6^,^9^,^14^,^27^.

The finding from Kelly & Fisher^12^, in women with normal voices, suggesting a reduction in ventricular fold action during inspiratory phonation supports the use of this technique in the clinical practice. Nonetheless, we still need much research in order to unveil the physiological mechanisms.

The inspiratory sound source is totally supraglottic, with a broad and inverse mucosal movement^15^. The technique may also be carried out during the utterance of vowels, syllables, words and phrases, performed from the low to the high frequencies and vice-versa, followed by its expiratory phonatory component^19^.

After learning the reverse phonation technique, the vowel /a/ may be pronounced with pitch variation, and such phonation may be practiced with other vowels, words, phrases, sentences, paragraphs and, during normal conversation^26^.

Once established, reverse phonation is quickly produced, followed by expiratory phonation. When the pitch of these two types of phonation become equal, the therapist can rest assured that the voice is starting to be produced by the true vocal folds. Little by little, reverse phonation must be eliminated and the voice starts being produced during expiration^26^.

In the study carried out by Balata^28^ about a case of psychogenic dysphonia, the inspiratory phonation vocal symptom installed during the phonotherapy. The author thought this vocal manifestation was very curious, the patient started to aspirate her voice, increasing her respiratory and vocal fatigue, making her speech intelligibility even more difficult. In the relevant literature of psychogenic dysphonias we did not find this type of manifestation, in which there was a reverse phonation establishment after its use during the therapeutic intervention.

Added to the use of this technique concerning voice, Kenjo^29^ reports the use of inspiratory phonation in the treatment of stuttering. After reverse phonation exercises, one may notice fluency improvement during expiratory phonation.

### Technique use as an aid in larynx assessment

Serafim^30^ reports that reverse phonation used during larynx assessment allows for a broad understanding of its structure, since the Morgani ventricles and the vestibular folds open up, allowing vocal fold mobilization, with clear glottic exposure, favoring the identification of small lesions, paralysis, among other pathologies. He also adds that in exams performed with expiratory uttering, the larynx is static, since glottis adduction muscles have characteristics which are similar to those of the expiratory muscles, making reverse phonation provide a dynamic study of the larynx, allowing for a more precise diagnosis, and consequently a more proper therapeutic intervention.

The ventricle and the deeper vocal fold layer must be flexible and tumor-free in order to answer to the different pressures developed during reverse phonation. When the ventricles and the supraglottis relax, vocal fold tumors may be ruled out. Reverse phonation is useful in the diagnosis of tumors in the piriform sinuses and the Morgani ventricles, since these structures relax and reveal tumors therein^8^.

Reverse phonation's greatest contribution, together with larynx endoscopy, to otorhinolaryngological diagnosis is the possibility of better defining the location of these lesions on the lamina propria layers, as is the case with cysts, which may be in superficial layers or strongly adhered to the lamina propria deeper layers^31^.

As previously mentioned, video-stroboscopy may give us precise information on the dynamic properties of benign larynx lesions. However, clinical experience tells us that larynx endoscopy, with or without stroboscopy, does not reliably tell us about the presence or the size of Reinke edemas. Thus, we suggest forced inspiration maneuvers during endoscopy in order to have a more precise and reliable diagnosis^32^.

### Using reverse phonation in lieu of physiologic phonation

It is clear that reverse phonation benefits patients with spasmodic dysphonia because of the psychological and acoustic voice changes it produces^12^,^16^. There is an improvement in speech fluency and intelligibility, and less discomfort during reverse phonation if compared to expiratory phonation^9^.

Despite the use of reverse phonation by individuals with spasmodic dysphonia in noisy environments be impaired, in noise-free environments, at the telephone or with the use of amplification in amphitheatre, reverse phonation is normal, fluent, and seems to be accepted by listeners, despite voice harshness^9^.

In general, difficulties are not found during long periods of reverse phonation, except when the individual is exposed to stress, nervousness or restricted time during speeches. Usually, these individuals have difficulties in coordinating speech and breathing. However, with time, the patient ends up developing strategies to compensate for these difficulties^9^.

Phonation using continuous, low pressure sounds, such as vowels, is easier to be produced in reverse phonation than the production of fricatives, of higher pressure, because of that it may be considered as a starting point for reverse phonation production^33^. They are also not difficult to be produced during reverse phonation, in an initial position, blunt explosive phonemes, such as /p/, for example. In these cases, the individual makes compensations in order to improve speech intelligibility, but is unable to fully compensate for these difficulties, making the listener to take into account the context and contrast in order to enhance understanding^9^.

Learning reverse phonation seems to require a change in diaphragm, abdominal and intercostals muscle control, as well as laryngeal muscle control. Learning this control does not seem to be difficult and may be carried out through exercises^9^.

## DISCUSSION

Contact reduction between vocal folds during reverse phonation may occur due to the involvement of inspiratory muscles, such as the crycoarytenoid muscle (CAP), which is activated during inspiration and is the primary abductor during speech^9^, and it is probable that it starts activity during reverse phonation^9^. The cricothyroid muscle (CT) is also activated during inhalation. During reverse phonation, the CAP and CT activities may antagonize the typically adductors during phonation, resulting in a smaller contact coefficient^12^.

The finding that there is a more significant reduction in vocal fold contact in men when compared to women may be explained by factors such as a more acute thyroid angle and the shorter and thinner vocal folds in women, which cause a reduction in mucosal wave amplitude, making the glottic signal noisier^12^.

Regarding arytenoid cartilage positioning, one may assume that a reduction in arytenoid compression, during reverse phonation, occurs together with a reduction in vocal fold adduction^12^. Moreover, we must stress the fact that women usually have a grade I triangular slit also during expiratory phonation ^34^.

During reverse phonation, the vocal folds close up and there is an increase in supraglottic pressure, favoring the Bernoulli phenomenon, since the mucosal vibration and the airflow happen towards the gravity force. Contrary to this, during expiratory phonation, the mucosa vibrates towards the gravity force and the airflow is against the gravity, impairing this phenomenon^10^,^33^.

This similar quality to the basal sound in reverse phonation is associated to an increase in laryngeal adduction^12^. Robb et al.^16^ reported that this fact suggests that vocal tract posture in reverse phonation is not stable.

An increase in glottic resistance caused by inspiratory air must be a factor that causes an individual with vocal motor control to increase vocal fold abduction during reverse phonation and, possibly, reduces the feeling of inspiratory effort^12^.

Many authors agree that there is a higher incidence of crying in reverse phonation during the neonatal period^13^,^34^, believing that such production is favored by flexible cartilage and ligaments which are characteristic of the neonate's larynx^35^ associated to a more anterior tongue positioning^36^, ^37^, ^38^.

There are many mechanisms involved in voice frequency modification, mainly: vocal fold stretching, mass and length during vibration. The more the vocal folds are elongated, the faster will be the glottic cycles and higher will be the frequency produced. On the other hand, the greater the vocal fold mass to be vibrated, the less glottic cycles will happen, lowering the frequency. Therefore, the greater the vocal fold tension, faster will be the cycles, higher the frequency will be^2^,^3^,^34^,^39^.

It is believed that this happens because of CT activity during reverse phonation, because it is activated during inspiration, and this may contribute to the increase in vocal fold pull^12^.

On the other hand, patients with spasmodic dysphonia have a reduction in fundamental frequency during reverse phonation^9^, probably because long periods of sound phonation also favor poor vocal quality that produces a pulsated voice /fry/ ^40^.

The acoustic differences identified between normal and reverse phonation may be explained if we consider the tongue's role during vowel uttering. Vowels /i/ and /u/ are articulated with a higher tongue positioning, and the /a/ involves a lower tongue positioning. The /a/ and the /u/ are articulated with the posterior tongue positioning, and the /i/ with its anterior positioning^16^.

The high and frontal positioning of the /i/ stabilizes the larynx, in such a way that, even with a change in phonation, the larynx remains in the same place. This fixation is acoustically reflected, because there are no significant changes in this vowel's fundamental frequency when we compare reverse phonation to normal phonation^16^.

In contrast, the lower and posterior tongue position during the /a/ phonation makes the vowel more prone to the reverse phonation demand, contributing to lowering the larynx, and thus, favoring a worsening in vowel production, both in men and women. This worsening is of about 140 Hz^16^.

Vowel /u/ is higher during reverse phonation and such finding is even more significant in women. The average fundamental frequency increase in women is of approximately 80 Hz; while in men it is of about 72Hz. It is believed that the production of /u/ in reverse phonation involves less contraction of the orbicularis labis, condition that favors an increase in fundamental frequency^16^. These findings are compatible with those from Miller et al.^41^, who studied frequency changes in vowels produced by men singers in three different situations: vocal fry, reverse phonation and singing. The authors concluded that vowels uttering during different forms of phonation depends on auditory and vocal tract postural control.

Clinical literature suggests intensity changes during reverse phonation^2^,^9^; however, it does not describe these changes in details^12^.

About the risks of using this technique, there is a consensus among authors as to the use of reverse phonation with caution, since it causes vocal tract drying when air is inhaled through the mouth, and also the risk of vocal fold lesions when produced in excess and associated with other vocal techniques^9^,^16^,^19^,^33^.

As to the use of this therapy, it has been observed that reverse phonation is used in psychogenic dysphonias due to the immediate change in muscle adjustment and vocal change alterations, since the emission that follows reverse phonation usually occurs in a low modal recording. In cases of glottis slits and vocal fold paralysis, it is used to cause vocal fold adduction^2^,^5^,^6^,^20^. In cases of aryepiglottic phonation, or ventricular phonation, reverse phonation reestablishes vocal fold vibration, and must have its use gradually reduced until phonation recovery^2^,^5^,^6^. Reverse phonation may be used in the treatment of stuttering, because the Valsalva maneuver, an airtight vocal fold closure, does not occur during inspiration, thus favoring fluence^29^,^42^.

Reverse phonation is used as an aid in the diagnosis of mass lesions, since it provides better visualization of laryngeal structures during its production^6^,^8^,^30^, ^31^, ^32^,^43^,^44^. The pro-gravity force arising from this increase in supraglottic pressure, associated with the Bernoulli phenomenon, causes even stronger vocal fold abduction, allowing edematous lesions to spread, making them different from stiffer lesions^31^.

Regarding physiological phonation being replaced by reverse phonation, in adduction spasmodic dysphonia, an increase in CAP action or the cricothyroid muscle (CT), during reverse phonation, may help stabilize the phonation, because of an unexpected blockage of the lateral crycoarytenoid (CAL) muscle action and that of the thyroarytenoid muscle (TA)^12^.

Although listeners consider the reverse phonation speech more intelligible and fluent, with a longer phonation time they consider it a rough vocal quality^9^. It is speculated that this roughness be due to a great variation in period and amplitude of the sound produced by reverse phonation. The low frequencies also contribute to a worse quality in the periods, which usually are long enough to extinguish the vocal tract energy between individual cycles, causing a pulsatile voice quality /fry/ ^40^.

What may explain fluency during reverse phonation is the fact that each inspiratory phase is usually associated with the action of different laryngeal muscles. Expiratory phonation is usually associated with the activity of the laryngeal adducting muscles, such as the TA, the CAL and the arytenoid muscle (A). Now, reverse phonation is marked by the action of the CAP, abductor muscle, associated with a simultaneous, or almost simultaneous, diaphragm contraction. It is improbable to have CAP's voluntary control to inhibit inspiratory contraction. Therefore, involuntary contraction during reverse phonation must respond to spasms through the laryngeal adducting muscles. This summation of adduction and abduction forces result in a smooth vocal fold approximation. ^9^

## CONCLUSION

The goals of the present investigation were clearly reached, since we could better clarify the aspects related to the physiologic changes that happen during reverse phonation, as well as its use in the speech therapy clinical practice.

In the literature we find numerous studies describing a considerable reduction in vocal fold contact coefficient during reverse phonation when compared to normal phonation. The growing apart of ventricular folds, the increase in fundamental frequency, the maintenance of intensity levels, the drop in subglottic pressure (causing ventricle distension) and the better defined piriform sinuses visualization are all reported.

Reverse phonation is present in the cry of neonates and it is also used in the clinical practice as rehabilitation technique, and it may also replace physiologic phonation in some patients with spasmodic dysphonia. In these subjects, it lowers the fundamental frequency and improves fluency. It has also been proven efficient in the therapeutic intervention of individuals with stuttering, improving their fluency. It is equally and successfully used in the otorhinolaryngological practice, aiding in the detection of vocal fold mass lesions during laryngoscopy.

Despite visible benefits, this technique must be used with caution, because it may cause vocal tract drying when air inhaling happens through the mouth. There is also the risk of causing lesions when performed in excess or associated with other techniques.

There are few studies that describe the larynx behavior during reverse phonation; therefore, to use this phonotherapy in a more precise and objective fashion, we still need objective studies to prove its efficacy in the phonotherapy clinical practice and to help us better understand its physiologic mechanism.
